# Foot pressure distribution during walking in young and old adults

**DOI:** 10.1186/1471-2318-5-8

**Published:** 2005-05-19

**Authors:** Mary Josephine Hessert, Mitul Vyas, Jason Leach, Kun Hu, Lewis A Lipsitz, Vera Novak

**Affiliations:** 1 Division of Gerontology, Beth Israel Deaconess Medical Center Harvard Medical School, Boston 02215 MA, USA

## Abstract

**Background:**

Measurement of foot pressure distribution (FPD) is clinically useful for evaluation of foot and gait pathologies. The effects of healthy aging on FPD during walking are not well known. This study evaluated FPD during normal walking in healthy young and elderly subjects.

**Methods:**

We studied 9 young (30 ± 5.2 years), and 6 elderly subjects (68.7 ± 4.8 years). FPD was measured during normal walking speed using shoe insoles with 99 capacitive sensors. Measured parameters included gait phase characteristics, mean and maximum pressure and force, and relative load.

Time-series measurements of each variable for all sensors were grouped into 9 anatomical masks.

**Results:**

Elderly subjects had lower normalized maximum pressure for the medial and lateral calcaneal masks, and for all medial masks combined. In the medial calcaneus mask, the elderly group also had a lower absolute maximum and lower mean and normalized mean pressures and forces, compared to young subjects. Elderly subjects had lower maximum force and normalized maximum force and lower mean force and normalized mean forces in the medial masks as well.

**Conclusion:**

FPD differences between the young and elderly groups were confined to the calcaneus and hallux regions and to the medial side of the foot. In elderly subjects, weight bearing on the lateral side of the foot during heel touch and toe-off phases may affect stability during walking.

## Background

Measurement of foot pressure distribution (FPD) is clinically useful because it can identify anatomical foot deformities [1], guide the diagnosis and treatment of gait disorders and falls, as well lead to strategies for preventing pressure ulcers in diabetes. Age-related anatomical and physiological changes in foot bone and ligament structure affect FPD during gait [1]. Gait analysis of healthy elderly people has revealed decreased stride length, reduced step force and increased variability in gait parameters. These findings indicated that unsteadiness during walking is increased in the community-dwelling elderly people, posing a risk for falls [2]. Age was independently associated with lower pressure under the heel, midfoot, and hallux in the multivariate analysis [3]. Foot pressure studies during walking have focused on specific pathology and deformity [4-6] specific anatomical areas [7], exercise [8] and younger subjects [9-11]. Knowledge of the plantar FPD map during normal walking in healthy elderly people is lacking. It is not known if distribution of plantar pressure, force, and load across several anatomical regions of the foot during walking is different between young and old. To determine the effects of normal aging on FPD, we evaluated the anatomical distribution of plantar pressure, force, and relative loads in healthy young and old subjects during walking.

## Methods

### Subjects

The study was performed in the Syncope and Falls in the Elderly (SAFE) Laboratory at the Beth Israel Deaconess Medical Center, and all subjects signed informed consent approved by the Institutional Review Board. Subject characteristics are shown in the Table 1: 9 young subjects (5 men and 4 women; mean age 30 ± 5.2 years, mean ± SD, range 23–39 years), and 6 elderly subjects (4 men and 2 women, mean age 68.7 ± 4.8, range 63–74 years). All subjects were screened with a detailed medical history, physical activity questionnaire, electrocardiogram, and were not treated for any systemic disease. Inclusion criteria were: age range 20–40 or 60–80 years, ability to walk a flight of stairs, and ability to walk for 12 minutes. All subjects were normotensive by medical history, confirmed by measurements of the sitting and standing heart rate and blood pressure. Height and body mass were measured and used in FPD analysis. Shoe size and foot imprints were taken to determine the insole size. Exclusion criteria were: neurological or musculoskeletal abnormalities affecting gait, peripheral neuropathies, diabetes, past hip/leg/foot trauma or surgery, dementia, epilepsy, alcohol, smoking and other drug abuse, current pregnancy, history or physical evidence for coronary artery disease, abnormal electrocardiogram (ST-T wave changes, old myocardial infarction, arrhythmias, bundle branch block), history of more than one fall or syncope in the past year, orthostatic hypotension, systemic disease requiring continuous medical treatment for >1 month, malignant neoplasms, hepatic, renal, heart disease or failure, hypertension, and body mass index (BMI) >35. The arch indexes of the subjects were not measured.

**Table 1:** Demographic characteristics

**Demographics**	**Young**	**Old**	**P**
Age (years)	30 ± 5.2	68.7 ± 4.4	<0.0001
Men/Women	4/5	2/4	
Mass (kg)	72.3 ± 7.5	70.3 ± 13.6	NS
Height (cm)	175 ± 9.5	169.9 ± 7.8	NS
BMI	23.2 ± 4.6	24.2 ± 3.1	NS
NWS (m/s)	1.25 ± 0.25	1.2 ± 0.2	NS
Stride (s)	1.1 ± 0.2	1.04 ± 0.1	NS
Step (s)	0.6 ± 0.1	0.6 ± 0.1	NS
Stance (s)	0.6 ± 0.1	0.6 ± 0.1	NS
Swing (s)	0.5 ± 0.1	0.4 ± 0.04	NS
Rate Perceived Exertion (RPE)	2.5 ± 1.4	4.2 ± 1.2	0.004
Δ RPE Beginning-End	0.22 ± 0.7	0.1 ± 0.4	NS
Physical Activity Questionnaire	4.8 ± 1.1	4.9 ± 1.1	NS

Mean ± SD

NWS – normal walking speed

BMI – body mass index

### Experimental protocol and data acquisition

Foot pressure distribution was measured during walking on the treadmill at individual normal walking speed. A treadmill was used to ensure a consistent speed, despite of its artificial milieu, as plantar pressure and force vary at different gait speeds [6]. Self-paced normal walking speed (NWS) was determined with hallway walking for 12 minutes. During walking, the subject was followed by the investigator for safety and timing purposes. Total distance (m) was divided by the walking time to determine individual NWS. Gait characteristics at NWS (step, stride, stance and percentage of the initial and terminal double stance) were not different between the groups (Table 1). The treadmill walk started at 0.8 mph for all subjects and increased 0.2 mph every 30 seconds until NWS was achieved. All subjects walked at their NWS for 6 minutes. FPD analysis was done on 100 steps (50 left and 50 right) selected from steady data segments after 2 minutes of sustained treadmill walking. At the end of walking, the treadmill decelerated over 1-minute and stopped. Subjects rated perceived exertion using 10-point Borg Rating Perceived Exertion scale [12] before the treadmill walk started, at the end of the speed increase period, and at the end of normal walking. Subjects sat on a chair while heart rate and blood pressure were monitored for six additional minutes during the post-walk period. Foot pressure distribution was measured using the shoe insoles with 99 capacitive sensors, connected to a small portable data acquisition device that sampled pressure for each sensor at 50 Hz (Pedar Mobile, Novel Electronics Inc., GmbH Munich, Germany). The insoles were calibrated regularly using a 'Trublu' calibration device (Novel GmbH, Munich, Germany). Two insole sizes (size WW = European shoe size 40/41, size XW = European 41/42) were used to account for differences in foot sizes. To control for differences in personal footwear, all subjects were provided with a standard, thin pair of slippers.

### Data analysis

All data were visually inspected prior to analysis to assure high quality of data acquisition.

**Figure 1:**
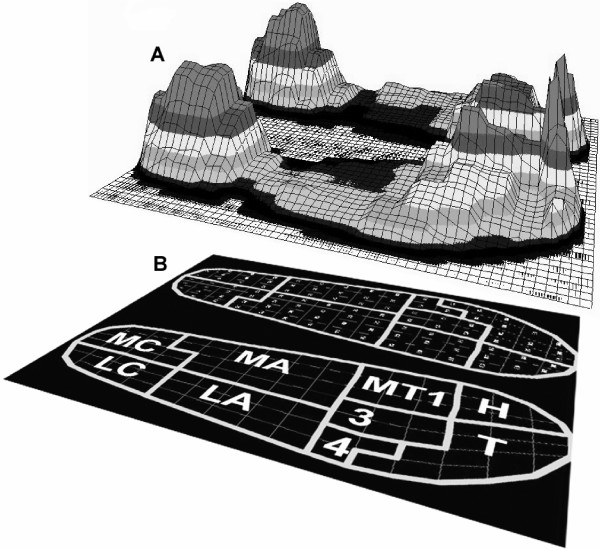
Foot pressure distribution. A. Maximum pressure distribution on all sensors during stance for one subject. B. The nine anatomical masks superimposed on the insole (MC = medial calcaneus, LC = lateral calcaneus, MA = medial arch, LA = lateral arch, MT1 = first metatarse, 3 = second and third metatarse, 4 = fourth and fifth metatarse, H = hallux, and T = toes).

Figure 1A shows distribution of maximum pressure for one step for all sensors. Time-series pressure measurements for all sensors were grouped into nine anatomical masks [5,13,14] (Figure 1B). These masks corresponded to the following anatomical areas: medial calcaneus, lateral calcaneus, medial arch, lateral arch, first metatarsal, metatarsals two and three, metatarsals four and five, hallux, and toes. The following 5 variables were calculated for the each mask: maximum pressure, maximum force, mean pressure, mean force, and relative load. All variables were calculated for each step and then averaged over the 50 steps for each foot. Maximum pressure was defined as the greatest pressure any single sensor in each mask measured in a single step, and these values were averaged separately for each mask over 50 steps. Mean pressure was defined as the average of all activated sensors in a mask for a single step. To calculate maximum and mean forces, the pressure time-series data were converted to force by multiplying each pressure value with the cross-sectional area of the corresponding sensor. All sensors in a defined mask were added together for each time frame to give the summed time-series for force, which was the total force for each mask. The maximum force was defined as the greatest force exerted for each mask in a single step. The mean force was defined as the average force exerted in each mask for a single step. Body weight was significantly different between men and women (p < 0.0001). All variables were normalized by body weight (BW) and the area of each mask, to account for these factors. Relative load was defined as the ratio of the total force in a specific mask to the total force of all masks combined, expressed as a percentage [5].

### Statistical analysis

The maximum and mean pressure and force were compared between the groups for all masks. In addition, we compared the mean and maximum pressure and force in the medial masks (medial calcaneus, medial arch, first metatarsal, and hallux) to the lateral masks (lateral calcaneus, lateral arch, second and third metatarsal, and toes) and between the groups. We also compared the anterior masks (hallux, toes, first metatarsal, second and third metatarsal, and fourth and fifth metatarsals) to the posterior masks (medial arch, lateral arch, medial calcaneus, and lateral calcaneus) and the anterior masks (hallux and first metatarsal) similarly. Within group comparisons between masks were done using nonparametric ANOVA (Friedman test). Comparisons between groups were done using Wilcoxon nonparametric test using JMP 5.0.1 software (SAS Institute 2003).

## Results

**Figure 2:**
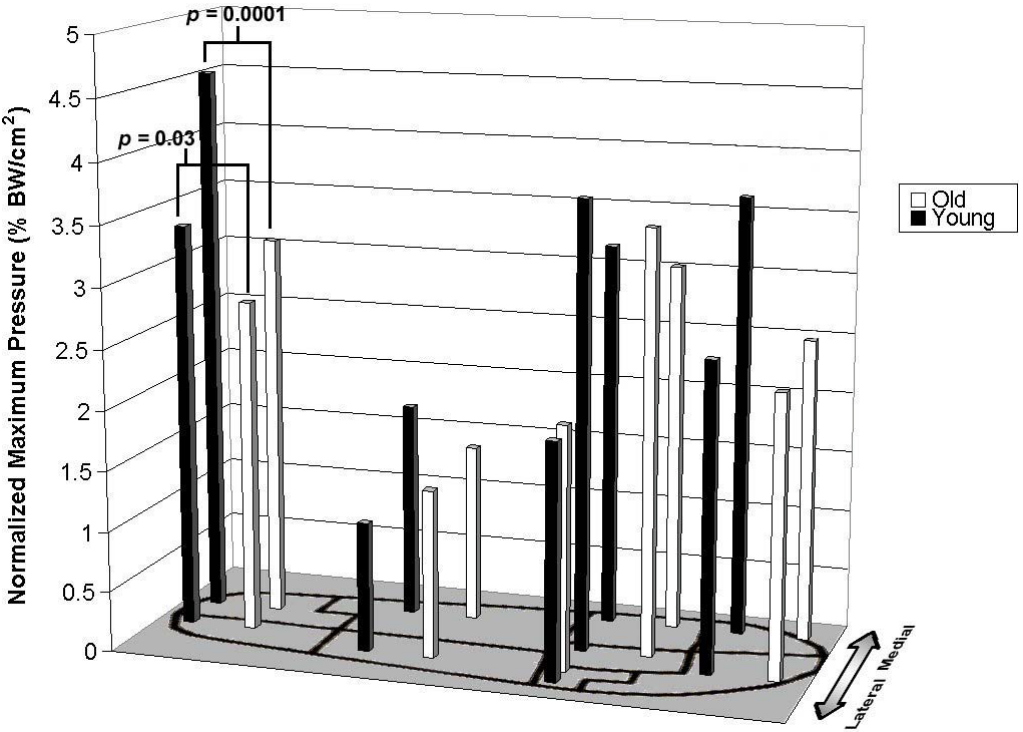
Pressure distribution by anatomical region. Normalized maximum pressure distribution for the young (white bar) and elderly (black bar) group for each anatomical region (medial calcalneus mask p = 0.0001, lateral calcaneus mask p = 0.03).

**Figure 3:**
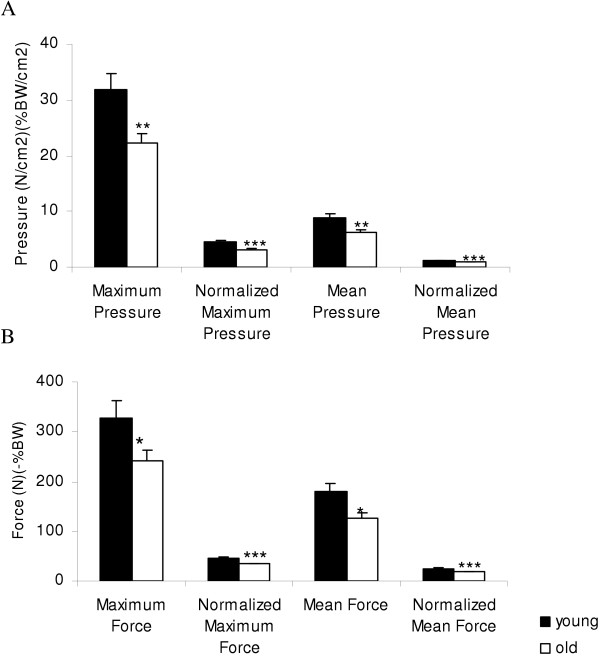
Medial calcaneus mask. A. The average maximum and mean pressures, and normalized mean and maximum pressures for medial calcaneus mask for young and old subjects (**p = 0.01, *** normalized maximum pressure p = 0.001, *** normalized mean pressure p = 0.0006). B. The average mean and maximum forces and normalized mean and maximum forces for medial calcaneus for the young and old groups (mean ± SD, maximum force * p = 0.05 *** normalized maximumn force p = 0.001, mean force * p = 0.02, *** normalized mean force p = 0.0006).

Foot pressure distribution was highly significantly different between masks for the young and old groups for all variables (maximum and mean pressures p < 0.00001, normalized maximum and mean pressures <0.00001 and maximum and mean force p < 0.00001 and normalized maximum and mean force p < 0.00001). Differences in the foot pressure distribution between the young and old groups for the maximum and mean pressures were confined to the calcaneus region and to the medial masks of the foot. Figure 2 shows differences in maximum pressure distribution (normalized for body weight (BW)) for all 9 anatomical regions. Elderly subjects had lower normalized maximum pressure in the medial (3.2 ± 0.5 vs. 4.6 ± 1.1 %BW, p = 0.001) and lateral (2.8 ± 0.6 vs. 3.4 ± 0.8 %BW, p = 0.036) calcaneal masks, for all medial masks combined (2.6 ± 0.2 vs. 3.3 ± 0.2 %BW/cm^2^, p = 0.019) and marginally reduced in the hallux mask (2.5 ± 1.2 vs. 3.7 ± 1.6%BW/cm^2^, p = 0.07). In the medial calcaneus region, the elderly group also had lower maximum pressure (22.2 ± 6.3 vs. 32.9 ± 11.8 N/cm^2^, p = 0.01), as well as lower mean (6.2 ± 1.8 vs. 8.9 ± 3.1 N/cm^2^, p = 0.01) and normalized mean pressure (0.9 ± 0.2 vs. 1.2 ± 0.2 %BW/cm^2^, p = 0.0006) (Figure 3A). The elderly subjects had also lower maximum force (240.9 ± 77.9 vs. 328.4 ± 138.7 N, p = 0.049) and normalized maximum force (34.3 ± 6.2 vs. 45.6 ± 8.5 %BW, p = 0.001) and the mean force (126.3 ± 34.9 vs. 178.8 ± 71.0 N, p = 0.02) and the normalized mean forces (18.3 ± 3.5 vs. 24.6 ± 4.2 %BW/ cm^2^, p = 0.0006) (Figure 3B). In the medial region masks, the elderly group had reduced maximum pressure (17.7 ± 1.6 vs. 23.2 ± 1.4 N/cm^2^, p = 0.02), reduced normalized maximum pressure (2.6 ± 0.2 vs.3.3 ± 2.0 %BW, p = 0.019); and reduced normalized mean pressure (0.5 ± 0.1 vs. 0.7 ± 0.1 % BW, p = 0.037) and borderline mean pressure (3.5 ± 0.5 vs. 4.8 ± 0.4 N/cm^2^, p = 0.06). In all anterior region masks, the elderly group exerted lower mean pressure (4.1 ± 2.3 vs. 4.8 ± 2.2 N/cm^2^, p = 0.046), borderline reduced mean force (57.3 ± 36.0 vs. 67.2 ± 33.6 N, p = 0.052) and borderline reduced normalized mean force (8.2 ± 4.5 vs. 9.8 ± 5.0 %BW, p = 0.055).

In the anterior medial region (hallux and first metatarsal), the elderly group displayed reduced mean pressure (3.6 ± 2.2 vs. 4.7 ± 2.1 N/cm^2^, p = 0.03), reduced normalized mean pressure (0.5 ± 0.03 vs. 0.7 ± 0.3 % BW, p = 0.027). The relative load in the medial masks (11.0 ± 8.8 vs.12.0 ± 7.8%) and the lateral masks (11.2 ± 6.8 vs.10.4 ± 6.2%) were not different. The relative load over the medial and lateral arches was not different between groups. Elderly subjects lower medial pressure values compared to young subjects, indicates that older people had tendency for greater weight bearing on the lateral mask relative to young subjects. The arch height, has not been measured, however, the contact mid-foot area was not different between the groups.

Rate perceived exertion was higher in the older subjects at the end of treadmill speed increment (beginning of normal walk) (p < 0.01), as well as at the end of normal treadmill walking (p < 0.004). However, the difference in perceived exertion between the beginning and end of normal walk was not significant between groups. There was no significant difference between groups in the physical activity questionnaire on the scale 0–10 (Table 1).

## Discussion

Our study has shown that elderly people exert less pressure and force under the medial masks of the foot (medial calcaneus, hallux, anterior and posterior medial masks) during heel touch and toe-off phase. This implicates that elderly subjects preferentially bear weight on the lateral foot during normal walking. Lateralization of foot pressure suggested that medial weight bearing from heel-strike to toe-off is limited in older people compared to younger subjects. Well-distributed weight bearing and foot pressure compensate for the forces and heavy loads imposed on the foot during normal walking. Treadmill walking is different than normal walking due to an inability to change speed voluntarily and reduced stride variability. Although an artificial pace and walking environment are imposed by use of a treadmill, it was a tool used to maintain experimental control. Because foot pressure distribution is affected by walking speed and stride variability, [6] it was deemed necessary to control the speed using treadmill walking. In the posterior masks, the older subjects exerted lower maximum pressure and force on the calcaneus region when normalized for body weight, indicating that, along with the results above, maximum pressure at heel strike is also lower in old subjects than in young subjects. These findings may indicate that forces needed to stabilize the ankle during heel touch phase are reduced in older people. In the anterior masks, the elderly subjects also exerted lower normalized mean pressure and lower normalized mean force compared to young subjects. These findings, supported by results in the hallux mask, support the notion that old subjects have lesser ability to push-off in anticipation of the swing phase.

Walking may present a challenge to elderly people, and several age-related gait changes have been identified [2,3]. Morag et al. 1997[3] found that age correlated with heel pad stiffness, but to a lesser degree with walking speed, soft tissue characteristics, and height of the medial longitudinal arch. Our study did not confirm an age-related arch-flattening phenomenon to the extent of altered FPD, as forces in the medial arch area were not different between groups. Normal walking speed, stride intervals, timing within the gait cycle and the relative load in the arch area were similar between young and old subjects. These findings rebuke the notion that age-related decline in pressure is due to flattening of the longitudinal arch or that the stride length would be the primary factor underlying the reduced pressure and forces at heel strike. Anatomical foot structure, including soft tissue thickness and arch height, account for 35% of plantar pressure differences during gait [6]. Pressure values under the heel and midfoot are predominantly affected by weight bearing at the heel strike and midstance, whereas pressures in the anterior regions are determined to a greater extent by flexibility, muscle strength, and muscle recruitment [5]. Therefore, age-related soft tissue and bony structure degradation may reduce the capability of the plantar foot to deflect load [15]. FPD pattern in older people was similar to the pattern of experimentally reduced plantar sensation by cooling, emphasizing that decline in proprioception with aging may contribute to these results [11]. Older runners exhibited significantly more knee flexion at heel strike, but the range of motion and peak maximal vertical forces were reduced. The ground impact force and the initial rate of loading at heel strike were greater, indicating loss of shock absorbing capacity in older people [8].

### Limitations

The number of subjects in our study was relatively small, but was comparable to other studies of gait and foot pressure [5,9]. However, our study used the large number of steps during steady walking compared to previous studies. Moreover, selection of steps from the middle portion of the walk minimized inconsistencies that may accompany gait initiation and termination [5]. The foot shape (high vs. flat arch) has not been assessed. However, the midfoot contact area and the relative load in the medial and lateral arch masks were not different between groups.

## Conclusion

Healthy aging affects the dynamics of foot pressure distribution during normal walking. The forces and pressures under the medial foot masks were reduced in elderly people, resulting in lower propulsion during the step from the heel-touch to the toe-off phases. Clinically, lateralized foot pressure and lessened propulsion may affect walking ability in elderly people, posing difficulties in balance, forward thrust, and terrain adaptation.

## List of abbreviations

BMI = body mass index

BW = body weight

FPD = foot pressure distribution

NWS = normal walking speed

## Competing interests

The author(s) declare that they have no competing interests.

## Authors' contributions

MH – the recipient of American federation on Aging Scholarship, designed and performed data analysis and wrote the first version of the manuscript. MV – participated in the experiments, software development and analysis. JL – participated in the experiments and analysis. KH – participated in the statistical analysis. LL – contributed to data interpretation and manuscript preparation. VN – designed the study, conducted the experiments, participated in the analysis, data interpretation, and manuscript preparation.

## Pre-publication history

The pre-publication history for this paper can be accessed here:


